# Evaluating the Impact of Oral Contraceptives on Pancreatic Cancer Risk: A Two-Sample Mendelian Randomization Analysis

**DOI:** 10.3390/biomedicines13061351

**Published:** 2025-05-31

**Authors:** Yuxin Tang, Yu Zhang, Shuaiyi Wang, Xinyi Shi, Xinjia Ruan, Yu Cheng, Fangrong Yan, Tiantian Liu

**Affiliations:** School of Science, China Pharmaceutical University, Nanjing 210009, China; 3223051531@stu.cpu.edu.cn (Y.T.);

**Keywords:** oral contraceptive (OC), pancreatic cancer (PC), mendelian randomization (MR), protein quantitative trait loci (pQTLs), causal inference

## Abstract

**Background:** The relationship between oral contraceptive (OC) use and pancreatic cancer (PC) risk remains controversial, with inconsistent findings reported in observational studies. To clarify this relationship and better identify potential risk factors for PC prevention, more unbiased and robust approaches are needed. **Methods:** We investigated the potential causal relationship between OC use and PC risk using a two-sample Mendelian randomization (MR) analysis, with blood protein quantitative trait loci (pQTLs) as instrumental variables. To ensure the robustness of our findings, we performed a series of sensitivity analyses, colocalization analyses, and reverse MR. The causal effects of protein-coding genes on PC risk, as well as their expression patterns across different single-cell types, were subsequently investigated. To elucidate the potential pathogenic pathways, we conducted pathway enrichment analysis, protein–protein interaction (PPI) network analysis, and causal inference. **Results:** Our MR analysis identified five drug-targeted proteins significantly associated with PC risk. Higher levels of COMT, AGT, FN1, and UGT1A1, as well as lower levels of SERPINC1, were associated with an increased risk of PC. Among these, AGT, FN1, and COMT demonstrated consistent associations across sensitivity analyses and downstream analyses, providing robust evidence supporting their involvement in PC risk. **Conclusions:** This study provides genetic evidence suggesting, in European groups, a potential causal link between OC use and increased PC risk, possibly mediated through drug-targeted proteins such as AGT and FN1. These results highlight the importance for further research to elucidate the underlying mechanisms and assess the implications of OC use on PC risk.

## 1. Background

Pancreatic cancer (PC) is a highly aggressive and lethal malignancy, characterized by an alarmingly high mortality rate [1]. In the United States, the 5-year survival rate for PC remains approximately 10% [2], and its incidence is increasing annually by 0.5% to 1.0%. It is projected to become the second leading cause of cancer-related deaths by 2030 [3,4,5]. However, due to the absence of effective early diagnostic tools and preventive strategies, the public health burden of PC continues to grow. In recent years, the potential association between medication use and PC risk has garnered increasing attention. In particular, understanding how pharmacological agents may influence the development of PC through metabolic or hormonal regulation is critical for identifying risk factors and developing effective prevention strategies.

Oral contraceptives (OCs) are widely prescribed medications for both contraception and endocrine disorder management, particularly for patients with polycystic ovary syndrome (PCOS), where OCs are a common therapeutic option [6,7]. Studies have suggested that OCs influence cancer progression by altering the estradiol-to-progesterone ratio, modulating immune responses, and affecting one-carbon metabolism [8,9]. In recent years, numerous observational studies, including cohort and case–control designs, have investigated the potential association between OC use and PC risk. However, the results remain inconsistent: while some studies suggest that long-term OC use may increase PC risk [9,10], others indicate a potential protective effect [11,12], and some report no significant association at all [13]. These conflicting findings may result from various biases, including selection bias, information bias, and confounding by indication. The interpretation of the relationship between OC use and PC risk remains challenging due to the presence of multiple potential sources of bias. Given these discrepancies, there is a pressing need for more robust and rigorous approaches to assess the potential relationship between OCs and PC.

Mendelian randomization (MR) [14] offers a robust approach to address this issue. MR employs genetic variants as instrumental variables (IVs) to infer causal relationships between exposure factors and disease outcomes. Unlike conventional observational studies, MR minimizes the impact of confounding bias and reverse causation by leveraging the random assignment of genetic variants during conception. This method significantly enhances the validity of causal inference and has been extensively applied in pharmacological epidemiology to investigate drug–disease relationships [15,16,17,18,19,20,21]. Recent advancements in technology have led to significant breakthroughs in large-scale proteomics studies, particularly in the identification of protein quantitative trait loci (pQTLs), which have opened new possibilities for inferring the effect of plasma drug proteins on PC using a two-sample MR method [22,23].

This study aimed to investigate the potential causal relationship between OC use and PC risk. A two-sample MR analysis was employed to examine the relationship between OCs and PC risk at the protein level by identifying relevant drug targets as exposure. To strengthen the validation of the findings, colocalization and sensitivity analyses were conducted. Additionally, we conducted differential expression analysis to validate the gene expression differences of drug target genes in pancreatic cancer tissues compared with normal pancreatic tissues. Furthermore, we integrated single-cell RNA sequencing data with enrichment analyses to identify the specific cell types enriched for drug target genes in pancreatic tissues, as well as the biological pathways through which these genes may influence pancreatic cancer progression. These comprehensive analyses provide valuable insights into the potential impact of OC use on PC risk.

## 2. Methods

### 2.1. Overall Design

**Figure 1:**
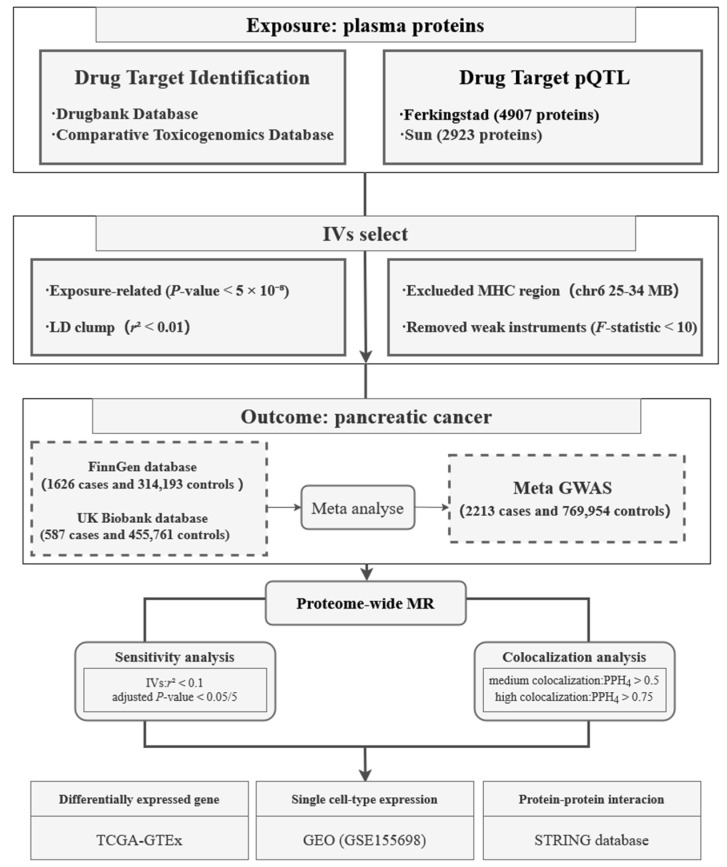
Flowchart of the study design. pQTL, protein quantitative trait loci; IV, instrumental variable; LD, linkage disequilibrium; MHC, major histocompatibility complex; GWAS, genome-wide association study; MR, Mendelian randomization; GEO, Gene Expression Omnibus.

This study was based on publicly available summary-level datasets, and the overall study design is illustrated in Figure 1. Briefly, the study was divided into three major components: In the first part, we employed pQTLs of drug-targeted proteins as potential instrumental variables, with PC as the outcome for MR analysis. The second part involved a series of sensitivity analyses and Bayesian colocalization analyses to validate the robustness and specificity of the MR findings. In the third part, we conducted differential expression analysis at the bulk-tissue level, investigated the cell type-specific expression of target genes using single-cell transcriptomic data, and explored their functional interactions through a protein–protein interaction (PPI) network analysis. All reported *p*-values were adjusted for multiple testing by the Benjamini–Hochberg (BH) procedure.

### 2.2. Study Population and Datasets

We defined PC as a malignant neoplasm of the pancreas, and identified patient cases based on the disease codes ICD-10 C25, ICD-9 157, and ICD-8 157. Summary-level genome-wide association study (GWAS) data for PC risk were obtained from the UK Biobank [23] and FinnGen [24] cohorts; after meta-analysis, the combined dataset included 772,167 individuals of European ancestry, comprising 2213 PC cases and 769,954 controls.

In this study, plasma proteins were considered as the primary endpoints, with drug-targeted proteins serving as exposures. pQTL data for plasma protein levels were obtained from two large-scale proteomic studies: deCODE (covering 4907 proteins from 35,559 individuals) and UKB-PPP (covering 2923 proteins from 54,219 individuals). For each protein, only variants with a reference SNP ID (rsID) were retained. We selected instrumental variables for drug targets based on pQTL data from these two databases.

### 2.3. Identification of Drug Target Proteins

We included commonly prescribed OCs for analysis and categorized them into subtypes—including combined oral contraceptives, progestin-only pills, and emergency contraceptive pills—to minimize potential confounding factors and improve the precision of the association assessment. Drug targets were obtained using from the DrugBank database to ensure accurate identification of drug–protein interactions [25]. Given the widespread use and complex composition of combined oral contraceptives, we also considered additional proteins potentially affected by these formulations. To enhance coverage and ensure comprehensiveness, supplementary protein targets were retrieved from the Comparative Toxicogenomics Database (CTD) [26].

### 2.4. Selection of Instrumental Variables

To ensure the validity of MR, IVs must satisfy three core assumptions during selection: (1) the IVs are robustly associated with the exposure; (2) the effect of the IVs on the outcome is mediated only through the exposure variable; and (3) the effect of the IVs is not confounded by other variables. In this study, we defined the transcriptional start site of a protein-coding gene within 1 Mb as **cis**, and prioritized cis-pQTLs as IVs due to their direct relevance to protein expression. The following criteria were applied to screen for IVs: (1) Selection of single nucleotide polymorphisms (SNPs) commonly associated with the exposure (protein) as having genome-wide significance (*p*-value < 5 × 10^−8^) and with a minor allele frequency (MAF) > 0.01; (2) Independent SNPs were selected to reduce the risk of pleiotropy due to the linkage disequilibrium (LD) effect, with an LD threshold of *r*^2^ < 0.01, and the genetic distance was set at 10 Mb [27,28]; (3) SNPs within the major histocompatibility complex (MHC) region (chr 6: 25.5–34.0 Mb) were excluded due to their complex LD structure and high polymorphism; (4) For *R*^2^ and *F*-statistics, we used the following equations: *R*^2^ = 2 × MAF × (1 − MAF) × *β*^2^; *F* = *R*^2^ × (*N* − 2)/(1 − *R*^2^) [29], where the *F*-statistic measures the strength of the genetic instruments and *R*^2^ represents the proportion of variance in the exposure explained by the SNP. IVs with an *F*-statistic < 10 were removed to avoid weak instrument bias.

### 2.5. Mendelian Randomization

To ensure the scientific validity and robustness of the analysis, data preprocessing and harmonization were performed prior to the formal MR analysis. Specifically, we harmonized the protein IVs with PC meta-GWAS data, extracted overlapped IVs between the filtered exposure and the outcome datasets, and removed incompatible or palindromic SNPs. Subsequently, appropriate MR methods were selected based on the number of IVs available to estimate causal effects and assess potential pleiotropy. When only a single IV was available, the Wald ratio method was applied. For exposures with two or more IVs, the inverse-variance weighted (IVW) method was employed, as it is the most efficient approach under the assumption that all IVs are valid [30]. In cases with more than three IVs, the MR-Egger method was conducted to detect potential horizontal pleiotropy. If the MR-Egger intercept test results indicated no pleiotropy (*p*-value > 0.05), the IVW method was adopted. Otherwise, causal inference was based on MR-Egger results. Heterogeneity among IVs was assessed using Cochrane’s Q-test. When both the UKB and deCODE datasets provided significant results, the dataset with a greater number of IVs was selected for causal estimation. All MR analyses were conducted using the “TwoSampleMR” R package [31].

### 2.6. Sensitivity Analysis

MR results can be influenced by the selection of IVs. In the sensitivity analysis, we re-selected the IVs using a relaxed LD threshold of *r*^2^ < 0.1. Although using a larger set of IVs would increase statistical power, it can also introduce potential confounding effects [16,32]. Therefore, it is essential to ensure that the causal direction observed in the sensitivity analysis remained consistent with that of the primary MR analysis. To control for multiple testing, the Bonferroni correction was applied to identify significant associations. Furthermore, to minimize the risk of potential reverse causality bias, we conducted a reverse causality analysis by using PC as the exposure and cis-pQTLs as the outcome. For selecting IVs for PC, we set a *p*-value threshold of <5 × 10^−8^ and an *r*^2^ < 0.01 within a 10 MB window to ensure the validity of the IVs.

### 2.7. Colocalization Analysis

To further validate the results, we performed a Bayesian colocalization analysis, which estimates the posterior probability that a given genomic locus contains a single causal variant influencing both protein levels and disease risk. This approach helps assess whether the observed associations between drug target proteins and PC risk are confounded by LD. For each protein, we selected independent cis-pQTL within a 500 kb window as the candidate colocalized regions. The analysis evaluates five hypotheses: (1) H_0_: no causal variant for either drug target protein or PC; (2) H_1_: a causal variant only for protein; (3) H_2_: a causal variant only for diseases; (4) H_3_: two different causal variants for protein and diseases, respectively; and (5) H_4_: one shared causal variant influencing for both traits. The posterior probability (PP) for each hypothesis is denoted as PPH_0_, PPH_1_, PPH_2_, PPH_3_, and PPH_4_ [33].

For proteins with multiple independent loci, colocalization analysis was conducted separately for each locus using meta summary-level data. Regions with the highest posterior probability of H_4_ (PPH_4_) were considered to provide the strongest evidence for colocalization. We defined medium-support evidence of colocalization as a PPH_4_ between 0.50 and 0.75, and high-support colocalization as a PPH_4_ > 0.75 [34]. Bayesian colocalization analysis was conducted using the ‘coloc’ package.

### 2.8. Differentially Expressed Genes

To further validate the MR findings and explore whether the causal targets identified were differentially expressed in PC, we conducted a differential expression analysis between pancreatic tumor and normal pancreatic tissues. RNA transcriptomic data were obtained from 178 pancreatic cancer samples in The Cancer Genome Atlas (TCGA) “https://www.cancer.gov/tcga” (accessed on 19 September 2024) and 167 normal pancreatic tissue samples from the Genotype-Tissue Expression (GTEx) project. Normalized data were retrieved from the UCSC Xena platform [35]. Genes were defined as differentially expressed (DEGs) if they exhibited a Log_2_ fold change (Log_2_ FC) > 2 and an adjusted *p*-value < 0.05. Differential expression analysis was conducted using the Limma package to identify DEGs that met these criteria [36]. This analysis provided additional evidence supporting the causal relevance of the drug targets implicated by MR.

### 2.9. Single Cell-Type Expression Analysis

Single-cell RNA sequencing (scRNA-seq) provides valuable insights into the cell type-specific effects of drug targets and the underlying pathways involved in the development of PC. We analyzed scRNA-seq data derived from human pancreatic tumor tissues and adjacent normal tissues, available from the Gene Expression Omnibus (GEO) database [37] (GSE155698) [38,39]. This dataset included 16 pancreatic cancer samples and 3 adjacent normal samples. Prior to analysis, rigorous data preprocessing was performed to ensure high data quality for downstream applications. Specifically, using the “Seurat” package, we applied the following quality control criteria: retention of cells with gene counts between 600 and 4000, total RNA count per cell greater than 1000 but below the 97th percentile of the dataset, mitochondrial gene content below 10%, and hemoglobin gene percentage below 1%. After filtering, 23,432 cells and 24,725 genes were retained for analysis. Gene expression values were normalized and scaled using the NormalizeData and ScaleData functions in Seurat. Cell clustering was performed, and clusters were manually annotated based on canonical marker genes to facilitate cell type-specific downstream analyses.

To investigate the role of genes encoding target proteins in specific cell types, we performed a differential expression analysis between pancreatic cancer and adjacent normal tissue cells within each cell cluster. This analysis was conducted using the Wilcoxon rank-sum test, implemented in the FindMarkers function in Seurat. Genes were considered differentially expressed if they exhibited an average |Log_2_ FC| > 1 and an adjusted *p*-value < 0.05. Subsequently, we performed Gene Ontology (GO) and Kyoto Encyclopedia of Genes and Genomes (KEGG) enrichment analyses on the identified differentially expressed genes. Particular attention was given to the biological process (BP) category in the GO enrichment to elucidate the potential biological functions and mechanisms underlying the cell type-specific DEGs [40].

To further explore the cell type-specific expression of genes encoding pathogenic proteins within pancreatic cancer tissues, we performed differential expression analysis across single-cell clusters. Specifically, we compared gene expression levels between different cell types using the Wilcoxon rank-sum test. Genes with an average |Log2 FC| > 1 and an adjusted *p*-value < 0.05 were considered significantly enriched in specific cell types.

### 2.10. Protein Interaction Networks and Protein Function Queries

To explore the known functions and biological roles of the target proteins, we utilized the STRING database https://string-db.org/ (accessed on 26 December 2024) and conducted a comprehensive literature review. This approach enabled the exploration of protein–protein interactions (PPIs), the identification of potential functional associations, and the recognition of key molecules involved in relevant biological processes. By integrating evidence from both experimental studies and computational predictions, this provided valuable insights into the involvement of these proteins in cellular mechanisms and disease pathophysiology.

## 3. Results

### 3.1. MR Results Identified PC-Related OC Target Proteins

In this study, the oral contraceptives investigated include ethinyl estradiol, levonorgestrel, desogestrel, cyproterone acetate, medroxyprogesterone acetate, dienogest, estradiol valerate, gestodene, spironolactone, finasteride, letrozole, and drospirenone. Drug-related targets were identified using the DrugBank database, resulting in 118 drug-related targets. Additionally, combined oral contraceptives, which are defined as compound prescriptions containing estrogen and progesterone, were also considered, and 48 interaction targets were retrieved from the CTD. Detailed drug target information is provided in Supplementary Tables S1 and S2. After applying thresholds for *p*-values and *r*^2^, we calculated *F*-statistics, with all genetic instruments having *F*-statistics > 10. Following the selection of IVs, a total of 319 SNPs were available to proxy 32 proteins in deCODE, and 256 SNPs were available to proxy 35 proteins in UKB-PPP.

**Figure 2:**
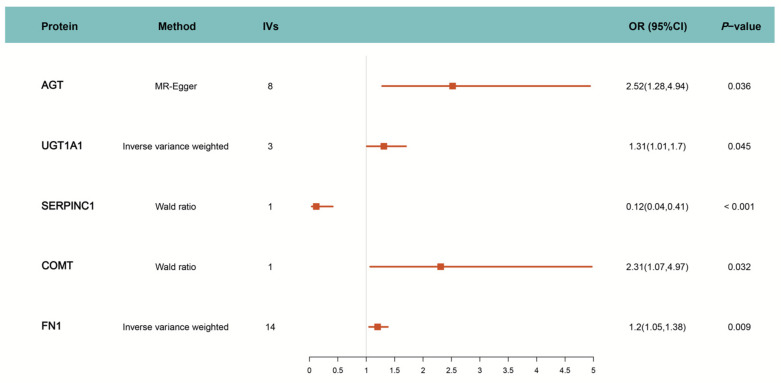
MR estimation of causal relationship between drug target and PC. IVs: Number of instrumental variables; OR: Odds Ratio; CI: Confidence Interval.

The primary analysis identified five proteins that were causally associated with PC at the blood level, as determined using IVW as the main MR method. These significant findings, along with the analytical approach, are illustrated in Figure 2. Specifically, the genetically predicted levels of four proteins (AGT, COMT, FN1, and UGT1A1) were positively associated with increased PC risk, while SERPINC1 was negatively associated with PC risk. Detailed information on the corresponding drug target interactions and their effects on PC risk are provided in Table 1. COMT and UGT1A1 are involved as substrates or inducers in the metabolism of hormonal drugs such as ethinyl estradiol, desogestrel, and estradiol valerate. In contrast, AGT, FN1, and SERPINC1 are known targets modulated by ethinyl estradiol [41] and combined oral contraceptives, including formulations co-administered with levonorgestrel, gestodene, norgestimate, and dienogest [42,43,44,45,46,47]. Notably, while ethinyl estradiol combined with dienogest has been reported to influence SERPINC1 activity [48], other COC formulations have been associated with either the upregulation or downregulation of these protein expression levels, depending on the specific drug combination and context.

### 3.2. Sensitivity Analysis and Colocalization Analysis

We conducted a series of sensitivity analyses to assess the robustness and reliability of our MR findings. First, the MR was repeated using additional sets of threshold-screened IVs (*r*^2^ < 0.1 within 10 Mb). The directions of causal effects for all initially significant proteins remained consistent with the primary MR result. To account for multiple testing, all *p*-values were adjusted using the BH procedure, and four targets (AGT, COMT, FN1, and SERPINC1) passed the corrected significance threshold of 0.01 (calculated as 0.05/5). It is worth noting that although UGT1A1 was significant in the primary MR analysis, its association did not remain significant after multiple testing correction (adjusted *p*-value > 0.05), likely due to the large number of comparisons. Accordingly, we prioritized the exclusion of targets—such as UGT1A1—that failed to pass multiple testing correction or exhibited signs of heterogeneity or directional pleiotropy. We further performed reverse MR analysis using 1401 IVs for PC, and found no significant evidence of reverse causality influencing drug target protein levels, suggesting that the observed associations were not biased by reverse causal effects. Moreover, no heterogeneity was detected using Cochran’s Q test, and no horizontal pleiotropy was observed through the MR-Egger intercept test. These analyses provide strong evidence that the MR results are both robust and reliable.

Despite the consistency across sensitivity analyses, a causal relationship between genetically determined protein levels and PC could still be influenced by LD, or it could reflect true shared causal variation between the two. To address these possibilities, we performed a colocalization analysis, with the results summarized in Table 2. Among the five candidate proteins, the PPH_4_ values for AGT (PPH_4_ = 0.93) and SERPINC1 (PPH_4_ = 0.99) showed high-support evidence for an association linked to a shared causal variant. Additionally, medium-support evidence of colocalization (PPH_4_ > 0.5) was observed for FN1 (PPH_4_ = 0.51) and UGT1A1 (PPH_4_ = 0.59) with PC. These results suggest a high likelihood of shared causal variants between AGT, SERPINC1, FN1, and UGT1A1 protein levels and PC risk, indicating that the MR findings for these proteins not attributable to LD. However, the absence of significant colocalization results does not undermine the validity of the MR analysis [49].

### 3.3. Identified Protein-Coding Genes Differentially Expressed Between Tumor and Normal Tissue

**Figure 3:**
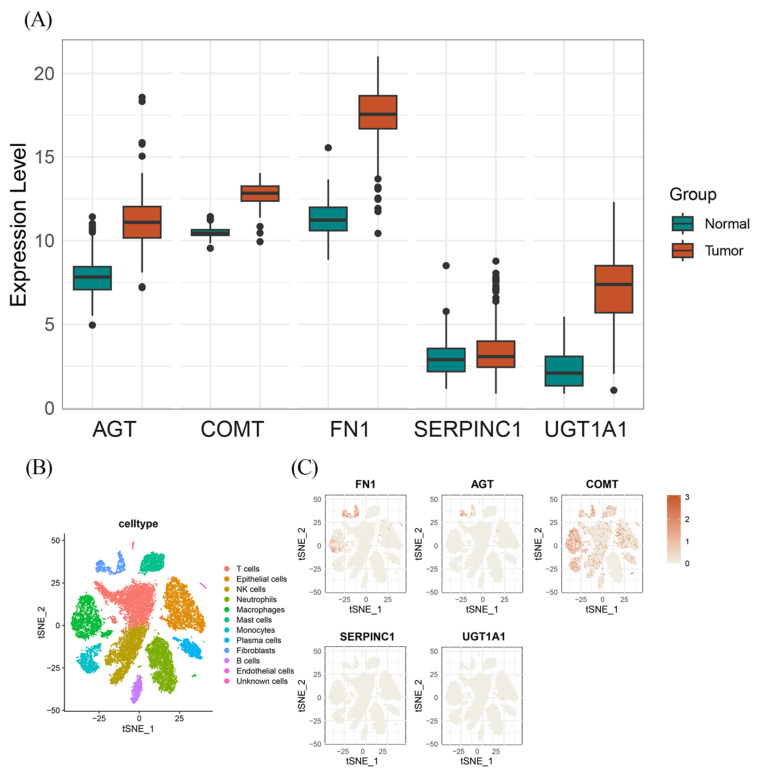
The results of DEGs and specific expression protein coding genes. (**A**) The DEGs in PC tissue for the protein-coding genes of the identified OCs; (**B**) Single cell-type expression in PC tissue represented the 11 cell clusters labeled and annotated; (**C**) The expression of identified protein coding gene in each cell type.

To investigate whether the identified protein-coding genes are differentially expressed in cancer and normal tissues, we conducted a comprehensive differentially expressed analysis. We applied a stringent correction for the FDR to control for multiple testing, with an adjusted *p*-value threshold of <0.05. The analysis revealed marked differences in expression levels for several target genes. Specifically, FN1 (Log_2_ FC = 6.2), UGT1A1 (Log_2_ FC = 6.1), AGT (Log_2_ FC = 3.31), and COMT (Log_2_ FC = 2.4) were significantly upregulated in tumor tissues compared to normal tissues. In contrast, SERPINC1 showed no significant differential expression, indicating that its expression levels remained relatively stable between tumor and normal samples. As shown in Figure 3A, nearly all of the identified protein-coding genes exhibited significant differential expression between tumor and normal tissues, with the notable exception of SERPINC1.

### 3.4. Single-Cell Type Expression in PC Tissues and Pathway Enrichment

To investigate the cell type-specific expression patterns of the identified protein-coding genes and their potential roles in PC development within distinct cellular contexts, we analyzed scRNA-seq data from 16 samples obtained from the GSE155698 dataset [38,39]. As shown in Figure 3B, all cells were manually annotated and classified into 11 clusters: T cells, epithelial cells, NK cells, neutrophils, monocytes, mast cells, plasma cells, fibroblasts, B cells, endothelial cells, and unknown cells. Cell type-specific gene expression profiles are shown in Figure 3C. After adjusting for *p*-value and performing the Wilcoxon rank-sum test, COMT was found to be ubiquitously expressed across all cell types, with the exception of monocytes. In contrast, AGT and FN1 were observed to exhibit highly cell type-specific expression patterns, with both genes predominantly expressed in fibroblasts compared to other cell clusters (Log_2_ FC values of 1.28 and 3.94, respectively). Additionally, FN1 exhibited notable expression in endothelial cells. No significant or specific expression of SERPINC1 or UGT1A1 was detected in any particular cell type in the single-cell dataset.

**Figure 4:**
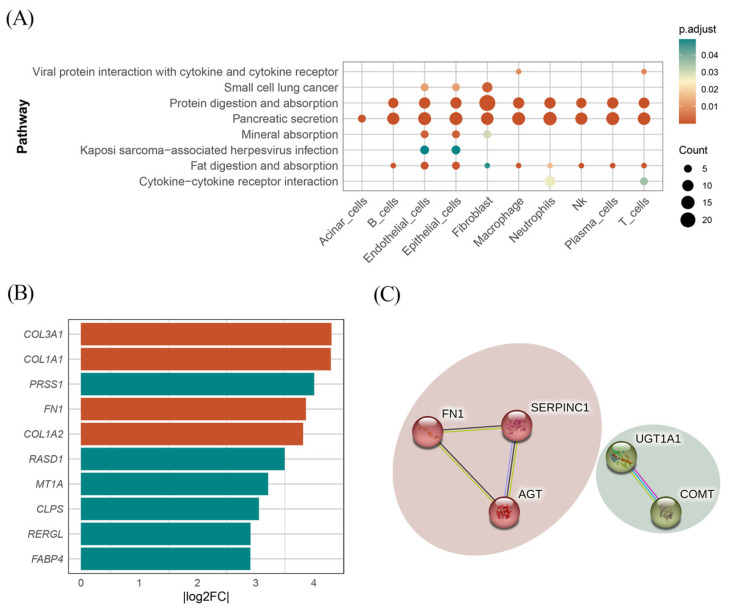
Results of KEGG and PPI network. (**A**) Cluster-specific DEGs KEGG pathway. (**B**) The top 10 DEGs identified in fibroblasts ranked by |Log2 FC|. Green indicates that Log2 FC is negative, and red indicates Log2 FC is positive. (**C**) Identified protein interaction network.

To further explore how the identified cell type-specific protein-coding gene influenced the progression of PC, we conducted a differential expression analysis between tumor and adjacent normal samples for each cell type, followed by KEGG and GO enrichment analyses. As displayed in Figure 4A, the KEGG analysis revealed that DEGs were broadly involved in pancreatic secretion pathways across all cell types. Additionally, protein digestion and uptake pathways were enriched in nearly all cell types, except for acinar cells. Focusing on the BP enriched within each cell type, we observed distinct functional signatures. In B cells, mast cells, macrophages, neutrophils, NK cells, plasma cells, T cells, and endothelial cells, DEGs were significantly enriched in processes related to digestion, leukotriene metabolism, regulation of lipase activity, lipid catabolism, and the positive regulation of lipase activity. Notably, fibroblasts exhibited a distinct enrichment pattern, with DEGs predominantly associated with structural and extracellular organization, including collagen fibril organization and extracellular matrix (ECM) organization. These pathways have been well-documented in pancreatic cancer progression, particularly in facilitating tumor growth, metastasis, and chemoresistance [38,50,51].

To further evaluate the involvement of the identified protein-coding genes in specific biological pathways, we calculated the Log_2_ FC of DEGs in fibroblasts. Among these DEGs, two identified genes (AGT and FN1) demonstrated Log_2_ FC values of 1.16 and 3.86, respectively. Notably, as shown in Figure 4B, FN1 exhibited an exceptionally high expression level, ranking fourth in terms of |Log_2_ FC| among all fibroblast DEGs. These findings suggest that FN1 and AGT may play critical roles in the progression of pancreatic cancer, particularly within the tumor microenvironment, and may represent promising therapeutic targets.

### 3.5. Protein PPI Network and Gene Function

To explore the interactions between the identified plasma proteins, PPI network analysis was performed using the STRING database. As shown in Figure 4C, with a confidence score of 0.4, interactions were observed between AGT, FN1, and SERPINC1, as well as between UGT1A1 and COMT.

To further examine the roles of the identified proteins in specific biological pathways, we investigated their known functions. COMT is involved in neurotransmitter metabolism, specifically degrading catecholamine transmitters through methylation [52]. AGT plays a critical role in maintaining fluid and electrolyte homeostasis and has been implicated in the pathogenesis of essential hypertension [53]. UGT1A1 participates in the glucuronidation pathway, which is essential for detoxification and the regulation of various substances in metabolism [54,55]. SERPINC1 is associated with coagulation, functioning primarily by inhibiting the activation of serine proteases and thrombin. Importantly, FN1 is crucial for cell adhesion and migration, playing a vital role in the ECM [56,57,58]; it contributes to the formation of structural fibers that facilitate tissue repair and supports the continuous remodeling of the ECM, thereby maintaining tissue strength and structural integrity [59].

## 4. Discussion

PC remains a major global health challenge due to its high mortality rate and limited improvements in survival, despite recent medical advances. This highlights the urgent need for effective preventive strategies [60]. In this context, identifying and mitigating risk factors, particularly the potential impact of OCs on PC, has become increasingly important. In recent years, numerous studies have investigated the relationship between OCs on PC risk. However, findings from observational studies have been inconsistent, largely due to inherent limitations such as selection bias, information bias, and confounding by indication [13,61,62]. These methodological weaknesses impede the ability to draw reliable causal inferences. Given these issues, using advanced analytical methods is essential to gain a more accurate understanding of the causal relationship between OCs and PC. MR offers a robust method of addressing confounding by leveraging genetic variants as instrumental variables, allowing for a more accurate assessment of the causal relationship between OCs and PC [63,64].

In this study, we systematically investigated the causal relationship between OC drug target proteins and PC risk using MR. Our analysis was structured into three kay sections, each addressing a specific objective: First, we aimed to identify drug target proteins that are causally linked to PC and evaluate the potential effects of OCs on these targets using MR. Second, we performed sensitivity analyses and colocalization analyses to assess the robustness of our MR findings. Third, we conducted additional validation through differential expression analysis, single-cell transcriptomic analysis, and enrichment analysis to explore the cell-specific expression of the identified protein targets and their potential biological functions, particularly in pathways relevant to PC progression. Our results identified key drug targets, including FN1, AGT, and COMT, that may mediate the effect of OCs on PC risk. Specifically, our findings suggest that OCs increase the expression of FN1 and AGT and utilize COMT as a substrate. FN1, a crucial component of extracellular matrix (ECM) organization, plays a pivotal role in cancer progression and metastasis [65,66,67,68,69,70,71,72,73]. Its upregulation in fibroblasts contributes to the fibrotic stroma of PC [74,75], thereby promoting tumor progression. Additionally, FN1 has been associated with drug resistance in PC and is considered a potential therapeutic target [76,77]. AGT has similarly been implicated in tumor growth in various cancers [78,79,80], and our findings further support its role in PC. Our findings suggest that co-treatment with different contraceptive drugs upregulates the expression of both FN1 and AGT, which may contribute to an increased risk of PC. Although Bayesian colocalization analysis for COMT showed negative results, both tissue and single-cell analyses indicated a strong correlation between COMT expression and PC. COMT overexpression has been linked to early-stage carcinogenesis in PC and is markedly elevated in PC tissues, correlating with features such as early T stage [81,82,83]. While the role of OCs in modulating COMT remains uncertain, evidence from animal models suggests that ethinyl estradiol can upregulate COMT expression, highlighting the need for further validation in human studies [84,85].

In conclusion, our study provides compelling evidence that OCs, particularly combined oral contraceptives, may increase PC risk by upregulating key drug target proteins, including FN1 and AGT. By leveraging MR to minimize confounding, we establish a robust causal relationship between OC use and elevated PC risk, supporting and extending findings from previous research. These results offer important insights into the underlying biological mechanisms of OC-associated PC risk. Further studies are warranted to validate these findings and to elucidate the precise molecular pathways involved.

However, several limitations should be considered: First, our analyses were limited to individuals of European ancestry, which may restrict the generalizability of the findings to other ethnic populations. Second, we focused on the pQTLs of drug-targeted proteins by leveraging the most comprehensive and up-to-date datasets currently available, aiming to ensure maximal coverage. However, we acknowledge that some relevant pQTLs may have been excluded, which may provide further insights into the pathogenesis of PC. Third, although the use of stringent criteria for instrument selection enhances validity, it may also reduce statistical power and potentially overlook relevant associations. For example, while UGT1A1 and SERPINC1 showed significant associations in the initial MR analysis, these findings were not supported by sensitivity analyses or DEG analyses. In addition, we did not include trans-pQTLs, which may have limited a more comprehensive understanding of the roles of these proteins in PC.

To address the limitations of our study, future research should prioritize the inclusion of ethnically diverse populations, particularly non-European groups, to improve the generalizability of the findings. Expanding the analysis to incorporate a wider range of pQTLs, including trans-pQTLs, may uncover additional drug targets involved in PC progression. Broader IV selection criteria and larger datasets will enhance the statistical power and may identify previously overlooked targets. Moreover, experimental studies are necessary to validate the regulation effects of OCs on COMT expression, especially in humans. Lastly, large-scale longitudinal cohort or case–control studies are also essential to confirm the causal relationship between OC use and PC risk, and to differentiate the effects of various OC formulations. Ultimately, these efforts will strengthen the evidence base for refining prevention strategies and therapeutic interventions.

In summary, our study utilized MR to investigate the causal effects of plasma drug targets associated with OCs on PC risk. Among the identified targets, FN1 and AGT demonstrated the strongest association, suggesting that combined oral contraceptives may contribute to increased PC risk through the modulation of these proteins. These findings suggest that oral contraceptives, particularly combined oral contraceptives, may influence the risk of PC development. However, further experimental and clinical validation is necessary to confirm these associations and clarify the underlying mechanisms.

## Figures and Tables

**Table 1:** Drug Target interactions.

Protein	Drug	Drug Target Interactions	Effect of the Protein on PC
COMT	ethinyl estradiol	substrate	Risk factors
AGT	ethinyl estradiol	increased expression	Risk factors
	ethinyl estradiol co-treated with levonorgestrel	increased expression	Risk factors
FN1	ethinyl estradiol co-treated with gestodene	increased expression	Risk factors
UGT1A1	ethinyl estradiol	substrate|inducer	Risk factors
	desogestrel	inducer	Risk factors
	estradiol valerate	inducer	Risk factors
SERPINC1	ethinyl estradiol co-treated with levonorgestrel	decreased expression	Protective factors
	ethinyl estradiol co-treated with norgestimate	decreased expression	Protective factors
	ethinyl estradiol co-treated with dienogest	decreased activity	Protective factors
	ethinyl estradiol co-treated with gestodene	decreased expression	Protective factors

**Table 2:** Sensitivity analysis and colocalization analysis.

	Primary Step MR		Sensitivity Step MR					Colocalization
Protein	OR	*p*-Value	OR	*p*-Value	Adjust *p*-Value	Revers Effect	Sensitivity Analyses	PPH_4_
FN1	1.20	0.0086	1.71	0.003	0.015	No	Passed	51%
COMT	2.31	0.032	1.15	0.001	0.005	No	Passed	21%
AGT	2.52	0.035	2.16	0.002	0.01	No	Passed	93.20%
UGT1A1	1.31	0.045	1.26	0.02	0.1	No	Failed	59.90%
SERPINC1	0.12	0.0007	0.12	0.0007	0.0035	No	Passed	100%

## Data Availability

All analysis was carried out in Rstudio 4.1.1. UKB-PPP pQTL https://www.synapse.org/Synapse:syn51364943/wiki/622119 (accessed on 12 December 2024). UKB PC GWAS (GWAS catalog: GCST90041814). FinnGen PC GWAS https://risteys.finngen.fi (accessed on 5 June 2024). deCODE pQTL https://www.decode.com/summarydata/ (accessed on 16 December 2024). GEO database: https://www.ncbi.nlm.nih.gov/geo/ (accessed on 26 December 2024). Single-cell data: GSE155698. GTEx-TCGA data: https://xenabrowser.net/datapages/ (accessed on 26 December 2024).

## Supplementary Materials

The following supporting information can be downloaded at: https://www.mdpi.com/article/10.3390/biomedicines13061351/s1, Table S1: drug target with Drugbank; Table S2: drug target with CTD.

## Abbreviations

PC	Pancreatic cancer
pQTL	Protein quantitative trait loci
Cis-pQTL	Cis-acting protein quantitative trait loci
MHC	Major histocompatibility complex
MAF	Minor allele frequency
GWAS	Genome-wide association study
UKB-PPP	UK Biobank Pharma Proteomics Project
MR	Mendelian randomization
LD	Linkage disequilibrium
IVs	Instrumental variables
SNP	Single nucleotide polymorphism
PPI	Protein–protein interaction
KEGG	Kyoto Encyclopedia of Genes and Genomes
STRING	Search Tool for the Retrieval of Interacting Genes
OR	Odds ratio
CI	Confidence interval
GEO	Gene Expression Omnibus
scRNA-seq	Single-cell RNA sequencing
